# Regulating the Practice

**Published:** 1895-07

**Authors:** 


### EDITORIAL DEPARTMENT.

F. E. DANIEL, M. D., Editor.
S. E. HUDSON, M. D., Managing Editor.
A. J. SMITH. M. D., Galveston, Associate Editor.

EDITORIAL STAFF:

PROF. J. E. THOMPSON, M. D., Texas Medical College, Galveston; Surgery.
PROF. WM. KEILLER, M. D., Texas Medical College, Galveston; Obstetrics and Gynecology.
PROF. DAVID CERNA, M. D., Texas Medical College, Galveston; Therapeutics.
PROF. A. J. SMITH, M. D., Texas Medical College, Galveston; Medicine
DR. R. H. L. BIBB, Saltillo, Mexico; Foreign Correspondent.

Official organ of the West Texas Medical Association, the Houston District Medical Association, the Austin District Medical Society, the Galveston County Medical Society, and several others

### REGULATING THE PRACTICE.

The JOURNAL is in receipt of "galley proofs of" Dr. Millard's article, entitled, "A Plea for Efficient Legislation Regulating Medical Practice." The paper was read before the American Academy of Medicine, at Baltimore, May 6, 1895, and was sent out to leading journals by the Secretary of the Academy, Dr. Charles McIntire, in obedience to a resolution adopted by the Academy, and with the request to "make as full use of it as possible, since its conclusions are expressive of the opinions of the Academy."

The subject has been discussed so much and so often, through the columns of the JOURNAL, both editorially and by subscribers, that we fear it is a little threadbare. But it is one of paramount importance, and the object sought *must be* accomplished. It is said that the constant dripping of a drop of water will, in time, wear away the hardest stone; and experience has taught that the best way to accomplish anything, is to keep pegging away at it; perseverance will win, in the long run, if the efforts be directed with judgment and discretion. It has been something of a hobby with the JOURNAL, and every argument in favor of throwing restrictions around the privilege of practicing medicine has been urged by us, time and again; but we are satisfied that the efforts at medical legislation in this State, all of which have been a failure, have not been put forward with the necessary judgment, however zealously pressed. In fact, we have always begun in the wrong way. As we pointed out in a recent article on this subject, the attempt has been made during the session of the legislature, at a time when members were occupied with other matters which they thought were of more importance, and it was difficult to get their attention. We suggested that the effort should begin long before the meeting of the legislature; even before members have been elected. Leading physicians—every physician who feels an interest in the welfare of his profession and who has a proper regard for the dignity of his calling—should make it a factor in the election; should canvas the subject with candidates for the legislature, convince them of the necessity of such legislation, and that it is not class legislation; enlist his interest and secure his pledge to support such a measure, before giving him their votes. Were this done, there can be no doubt of success. Members do not understand the subject; they must be enlightened, and the prejudices removed which, planted in their minds by the quacks and magnetic healers and other opponents of the bill, are deep-seated and hard to move.

In the hope, then, that Dr. Millard's paper may throw additional light on the subject, or stimulate some hitherto indifferent reader to an active interest, we have concluded to reproduce it in next issue, and comment on some of the main points and conclusions in the paper. The statistics are valuable, and should be preserved. We are the greatest nation of doctors and doctor-manufacturers on earth. The supply greatly exceeds the demand, and the works, most of them, should be shut down. The worst feature in connection with the situation is, other States "sift and sort" those turned loose on them, and the chaff—the refuse—flock to Texas, where there is no sorting machinery or winnowing process. We get all sorts. Let us be up and doing, even now.

